# The Role of N-Terminal-Pro-B-Type Natriuretic Peptide (NT-proBNP) and High-Sensitivity Troponin T (Hs-Troponin T) in the Evaluation of the Syntax Score in Patients With Acute Coronary Syndrome

**DOI:** 10.7759/cureus.55653

**Published:** 2024-03-06

**Authors:** Anh Binh Ho, Quoc Bao Tran

**Affiliations:** 1 Emergency - Interventional Cardiology Department, Hue Central Hospital, Hue, VNM

**Keywords:** cardiac markers, nt-probnp (n terminal-pro-b-type natriuretic peptide), high-sensitive troponin t, syntax score, acute coronary syndrome

## Abstract

Background

N-terminal-pro-B-type natriuretic peptide (NT-proBNP) is used to diagnose acute and chronic heart failure, but many studies show a strong and independent correlation between NT-proBNP serum levels and the severity and number of coronary artery damage. Meanwhile, the serum of high-sensitivity Troponin T (hs-Troponin T) has a very high prognostic value for the degree of coronary artery damage in patients with acute coronary syndrome. The SYNTAX score was developed to better predict the risks of percutaneous or surgical revascularization by considering the functional impact of the coronary circulation with all of its anatomic components, such as the presence of bifurcations, total occlusions, thrombus, calcification, and small vessels. Therefore, we conducted this study to understand the role of NT-proBNP and hs-troponin T in SYNTAX score evaluation in patients with acute coronary syndrome.

Methodology

A cross-sectional descriptive study of 86 patients diagnosed with acute coronary syndrome with indications for coronary angiography and intervention in the Department of Emergency and Interventional Cardiology, Cardiovascular Center, Hue Central Hospital, was conducted from June 2020 to May 2022.

Results

The mean age was 66.94 ± 10.61 years. The concentrations of NT-proBNP and hs-Troponin T in our study were 1115.9 ± 1623.3 pg/mL and 0.86 ± 1.55 ng/mL, respectively. The mean SYNTAX score in the study was 16.5 ± 7.5. There was a positive moderate correlation between the mean levels of NT-proBNP and the degree of coronary artery damage, as indicated by the SYNTAX score (*P* < 0.01, rho = +0.453). Conversely, there was a weak positive correlation between hs-Troponin T concentrations and the severity of coronary artery disease, based on the SYNTAX score (*P* < 0.01, rho = +0.387). The area under the curve (AUC) of the hs-Troponin T concentration value was 0.701, using a cutoff point of 0.109 ng/mL for hs-Troponin T concentration. This predicted the intermediate and high SYNTAX scores, with a sensitivity of 76% and a specificity of 59%. In comparison, the AUC of the NT-proBNP concentration value was 0.75, utilizing a cutoff point of 1120.5 pg/mL for NT-proBNP concentration. This predicted the intermediate and high SYNTAX scores, with a sensitivity of 60% and a specificity of 80.3%.

Conclusions

The levels of NT-proBNP had a positive moderate correlation with the degree of coronary artery damage according to the SYNTAX score in patients with acute coronary syndrome. Hs-Troponin T levels of 0.109 ng/mL had higher sensitivity (76%) but lower specificity (59%) in predicting intermediate and high SYNTAX scores in patients with acute coronary syndromes than those of NT-proBNP levels of 1120.5 pg/mL, with a sensitivity of 60% and a specificity of 80.3%.

## Introduction

Acute coronary syndrome (ACS) is a serious emergency event of coronary artery disease and a leading cause of cardiovascular death and serious complications [1]. According to the World Health Organization, 7.3 million people around the world die from coronary heart disease every year, accounting for about one-third of all causes of death in adults over 35 years of age [2]. The SYNTAX score was developed to better predict the risks of percutaneous or surgical revascularization by considering the functional impact of the coronary circulation with all of its anatomic components, such as the presence of bifurcations, total occlusions, thrombus, calcification, and small vessels. After getting the SYNTAX score, these scores were divided into three groups: groups with a low SYNTAX score of <23 points, intermediate SYNTAX score of 23-32 points, and high SYNTAX score of >32 points [3].

B-type natriuretic peptide (BNP) and N-terminal-pro-B-type natriuretic peptide (NT-proBNP) are used as laboratory tools to diagnose acute and chronic heart failure. In addition, BNP and NT-proBNP have been used as predictors of mortality and heart failure in patients with ACS and stable coronary artery disease. Many studies show a strong and independent correlation between serum NT-proBNP levels and the severity and extent of coronary artery damage according to the SYNTAX score, as well as the number of damaged coronary arteries [4,5].

Meanwhile, Troponin T is the marker commonly used to assess myocardial injury and is recommended as a biomarker for the diagnosis of acute myocardial infarction. The recent development of high-sensitivity Troponin T (hs-Troponin T) has shown improved accuracy in the diagnosis of myocardial infarction. Quantification of hs-Troponin T levels, which is recommended for routine clinical use, has a very high prognostic value for the degree of coronary artery injury in patients with ACS [6,7].

The importance of this combination of two biomarkers in the evaluation of coronary artery injury in ACSs according to the SYNTAX score needs to be further investigated. Therefore, we carried out this study to determine the correlation between serum NT-proBNP and hs-Troponin T levels and coronary artery damage in patients with ACS.

## Materials and methods

Data collection

This cross-sectional descriptive study enrolled 86 patients diagnosed with ACS who were indicated for coronary angiography and intervention at the Emergency-Interventional Cardiology Department, Cardiovascular Center, Hue Central Hospital, from June 2020 to May 2022.

Inclusion criteria were patients diagnosed with ACS according to the *Guidelines for Diagnosis and Management of Acute Coronary Syndromes* of the Ministry of Health 2019. ACS includes (1) acute myocardial infarction with ST-segment elevation (STEMI) on the electrocardiogram and (2) ACS without ST-segment elevation. ACS includes two types of presentation: non-ST-elevation myocardial infarction (non-STEMI) and unstable angina (UA). The patient volunteered and cooperated in the study process, or the patient's family provided consent for the patient's participation in the study.

The exclusion criteria encompassed patients with a previous history of heart failure due to structural heart disease or cardiomyopathy. Additionally, individuals with the following medical conditions, known to elevate hs-Troponin T, were excluded: muscle trauma, myocarditis, shock from any cause, renal failure, systemic disease, trauma, or cerebrovascular accident within the past three months. Participation in the study was not voluntary for both patients and their families.

Coronary angiography and SYNTAX score

Coronary angiography was performed under digital subtraction angiography (DSA) using the Judkins technique. The extent and complexity of coronary artery disease are assessed by various methods. The simplest way is based on the number of damaged coronary artery branches with stenosis ≥50%. In addition, the extent of coronary artery damage is also assessed using the SYNTAX score with lesions ≥50% stenotic in branches with a diameter of ≥1.5 mm. To calculate the SYNTAX score, we used the website: http://www.syntaxscore.com. After getting the SYNTAX score, these scores were divided into three groups: groups with a low SYNTAX score of <23 points, intermediate SYNTAX score of 23-32 points, and high SYNTAX score of >32 points [3].

Statistical analysis

Data were analyzed using IBM SPSS Statistics for Windows, Version 26.0 (IBM Corp., Armonk, NY). Quantitative variables were reported as mean and standard deviation if normally distributed, or median and interquartile range if not normally distributed; qualitative variables were reported by frequency and percentage and depicted in graphs. Significance was indicated by two-sided *P*-values < 0.05. A logistic regression model was used to examine the relationship between NT-proBNP and hs-Troponin T indices in comparison with the number of damaged coronary arteries. The Spearman correlation coefficient (rho) was used to consider the correlation between NT-proBNP and hs-Troponin T indices compared with the number of lesion sites or the number of damaged coronary arteries. The ROC curve was analyzed to determine the values of NT-proBNP and hs-Troponin T concentrations with the diagnosis of coronary artery damage according to the SYNTAX score.

## Results

The general baseline of the population

The study sample ranged in age from 42 to 92 years old, with an average of 66.94 ± 10.61 years. Nearly two-thirds had a history of hypertension. STEMI patients made up roughly half of the overall number of patients. The number of patients with one damaged coronary artery constituted more than one-third of the overall number of patients. The average SYNTAX score was 16.5 ± 7.5; low SYNTAX scores accounted for the highest percentage (Table 1).

**Table 1:** General characteristics of the population. HDL, high-density lipoprotein; STEMI, ST-elevation myocardial infarction; SD, standard deviation; ACS, acute coronary syndrome

Parameter	ACS cases ( *N* = 86)	
Age, mean ± SD (years)	66.94 ± 10.61 (42-92)	
Male, *n* (%)	30 (34.9%)	
Medical history, *n* (%)		
Hypertension	58 (67.4%)	
Diabetes	15 (17.4%)	
Dyslipidemia	38 (44.2%)	
Previous coronary intervention, *n* (%)	12 (14%)	
Smoking, *n* (%)	28 (32.6%)	
Diagnosis, *n* (%)		
STEMI	41 (47.67%)	
Non-STEMI	16 (18.6%)	
UA	29 (33.72%)	
Laboratories		Median (*N* = 86)
Glucose (mmol/L)	6.70 (5.64-8.45)	
Ure (mmol/L) or blood urea nitrogen (BUN)	5.6 (4.1-6.7)	
Creatinine (µmol/L)	72.9 (65.7-89.2)	
Total cholesterol (mmol/L)	5.15 (4.22-6.07)	
Triglyceride (mmol/L)	1.55 (1.23-2.22)	
HDL cholesterol (mmol/L)	1.12 (0.92-1.32)	
Number of damaged coronary arteries, *n* (%)		
1 vessel	30 (34.88%)	
2 vessels	37 (43.02%)	
3 vessels	19 (22.09%)	
SYNTAX score, *n* (%)		
Low (<23 points)	59 (68.60%)	
Intermediate (23-32 points)	25 (29.07%)	
High (>32 points)	2 (2.33%)	
Average SYNTAX score	16.5 ± 7.5	

Concentrations of NT-proBNP and hs-Troponin T in ACS

The concentrations of NT-proBNP and hs-Troponin T in our study were 1115.9 ± 1623.3 pg/mL and 0.86 ± 1.55 ng/mL, respectively. The average concentration of these two markers in the STEMI group was higher than that in the non-STEMI ACS group; this difference was statistically significant with *P* < 0.05 (Table 2).

**Table 2:** Concentrations of NT-proBNP and hs-Troponin T. NT-proBNP, N-terminal-pro-B-type natriuretic peptide; ACS, acute coronary syndrome; STEMI, ST-elevation myocardial infarction; hs-Troponin T, high-sensitivity Troponin T

	ACS	STEMI	Non-STEMI ACS	P
NT-proBNP (mean ± SD)	1115.9 ± 1623.3 pg/mL	1363.8 ± 1445.8 pg/mL	889.8 ± 1755.2 pg/mL	0.015
Hs-Troponin T (mean ± SD)	0.86 ± 1.55 ng/mL	1.31 ± 1.8 ng/mL	0.45 ± 1.17 ng/mL	0.01

Correlation between hs-Troponin T, NT-proBNP, and the degree of coronary artery lesion in patients with ACS

Relationship between hs-Troponin T, NT-proBNP concentrations, and coronary artery lesion characteristics (Table 3).

**Table 3:** Relationship between hs-Troponin T, NT-proBNP concentrations, and coronary artery lesion characteristics. NT-proBNP, N-terminal-pro-B-type natriuretic peptide; SD, standard deviation; hs-Troponin T, high-sensitivity Troponin T

*N* = 86		Hs-Troponin T (ng/mL)		NT-proBNP (mg/L)	
		Mean ± SD	*P*-value	Mean ± SD	*P*-value
Number of injured coronary artery	1 vessel (*n *= 30)	1.078 ± 1.839	0.514	878.17 ± 1168.95	0.558
	2 vessels (*n *= 37)	0.598 ± 1.201		1437.96 ± 2085.25	
	3 vessels (*n *= 19)	1.022 ± 1.674		863.92 ± 1082.27	
SYNTAX	Low SYNTAX (*n *= 59)	0.57 ± 1.19	0.011	837.1 ± 1567.6	0.001
	Intermediate SYNTAX (*n *= 25)	1.6 ± 2.1		1789.46 ± 1634.3	
	High SYNTAX (*n *= 2)	0.18 ± 0.08		805.65 ± 962.2	

There was no statistically significant difference between the number of damaged coronary arteries and NT-proBNP and hs-troponin T concentrations with *P* > 0.05. However, there was an association between these three markers and the SYNTAX score.

Correlation between hs-Troponin T, NT-proBNP, and coronary artery lesion characteristics according to the SYNTAX score (Table 4).

**Table 4:** Correlation between hs-Troponin T, NT-proBNP, and coronary artery lesion characteristics according to the SYNTAX score. NT-proBNP, N-terminal-pro-B-type natriuretic peptide; hs-Troponin T, high-sensitivity Troponin T

Markers	Acute coronary syndrome	
	Rho	P
NT-proBNP	0.453	<0.01
Hs-Troponin T	0.387	<0.01

There was a positive moderate correlation between NT-proBNP concentrations and the severity of coronary artery disease according to the SYNTAX score, with *P* < 0.01 and rho = +0.453, whereas there was a positive weak correlation between hs-Troponin T concentrations and the severity of coronary artery disease according to the SYNTAX score, with *P* < 0.01 and rho = + 0.387.

**Figure 1:**
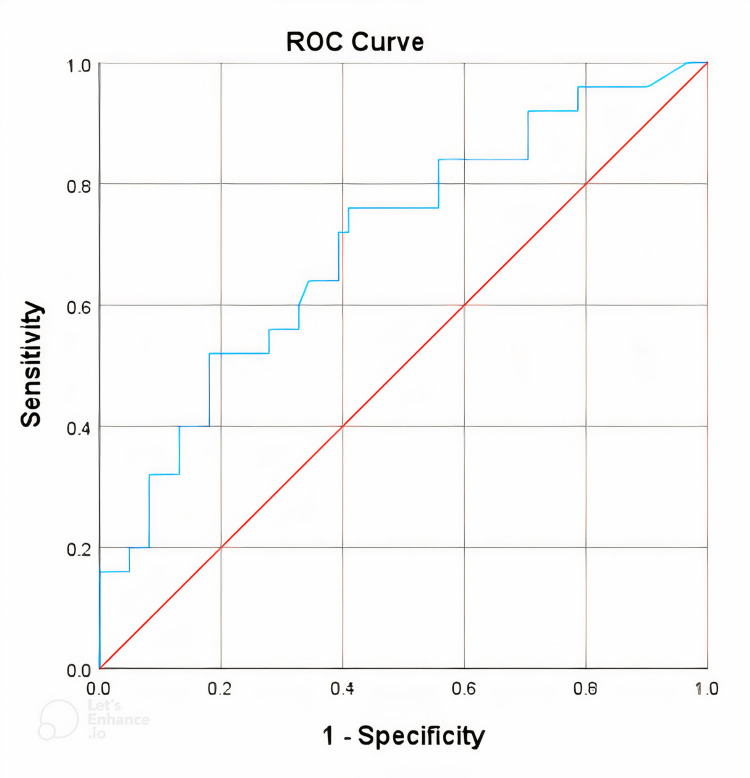
Diagnostic value of hs-Troponin T with intermediate and high coronary artery lesions according to the SYNTAX score. hs-Troponin T, high-sensitivity Troponin T; ROC, receiver operating characteristics

**Figure 2:**
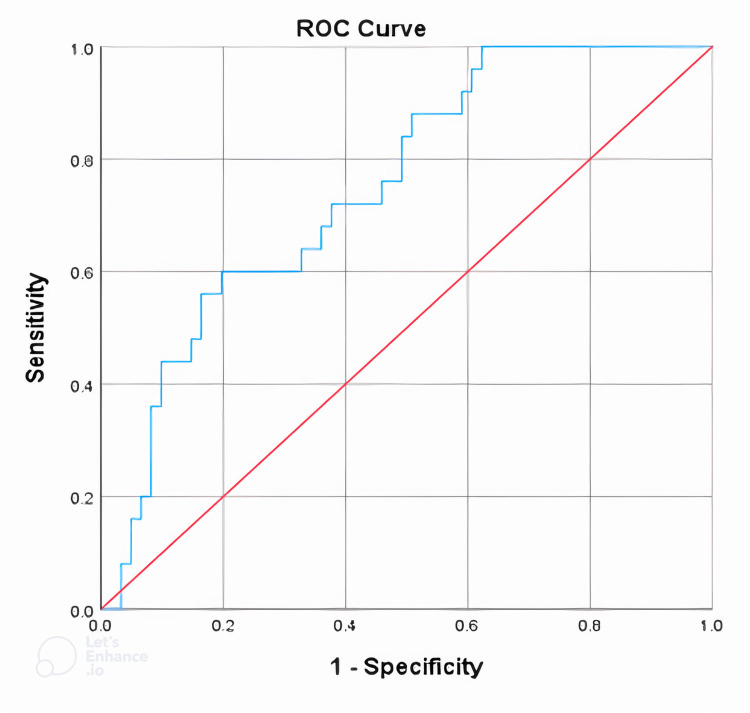
Diagnostic values of NT-proBNP with intermediate and high coronary artery lesions according to the SYNTAX score. NT-proBNP, N-terminal-pro-B-type natriuretic peptide; ROC, receiver operating characteristics

Concentrations of NT-proBNP and hs-Troponin T with the diagnosis of intermediate and high coronary artery damage according to the SYNTAX score (Figures 1-2).

The area under the curve (AUC) of the hs-Troponin T concentration value was 0.701, using a cutoff point of 0.109 ng/mL for hs-Troponin T concentration. This predicted the intermediate and high SYNTAX scores, with a sensitivity of 76% and a specificity of 59%. In comparison, the AUC of the NT-proBNP concentration value was 0.75, utilizing a cutoff point of 1120.5 pg/mL for NT-proBNP concentration. This predicted the intermediate and high SYNTAX scores, with a sensitivity of 60% and a specificity of 80.3%.

## Discussion

General characteristics of the population

In our study, regarding the diagnosis of hospital admission, the percentage of patients admitted to the hospital because of STEMI, non-STEMI, and UA was 47.67%, 18.6%, and 33.72%, respectively (Table 1). We documented that the group with equal to or more than two damaged coronary vessels accounted for the highest rate at 65.11% (Table 1), aligning with findings from other domestic and foreign studies [8-10]. This could be explained by the fact that, apart from the culprit lesions, most ACS patients could have more than two damaged coronary vessels. We recorded an average SYNTAX point of 16.5 ± 7.5, in which the low SYNTAX score group accounted for the highest percentage (59, 68.6%), followed by the intermediate SYNTAX score group (25, 29.1%), and the lowest was the high SYNTAX score group at 2 (2.3%; Table 1). Altun et al. recorded a low SYNTAX score of 42.5%, an intermediate SYNTAX score of 41.8%, and a high SYNTAX score of 15.7% [11]. A possible explanation is that the anatomy of damaged coronary vessels in ACS patients is intricate. The evaluation of the SYNTAX score is based on various anatomic components, including the presence of bifurcation, total occlusions, thrombus, calcification, and small vessels. These factors could lead to an increase in the SYNTAX score in ACS patients.

Concentrations of NT-proBNP and hs-Troponin T in evaluating coronary artery damage in patients with ACS according to SYNTAX scores

There was no statistically significant difference between the number of damaged coronary artery vessels and concentrations of NT-proBNP and hs-Troponin T with *P* > 0.05 (Table 3). However, there was a relationship between these two markers and the SYNTAX score.

NT-proBNP concentration and the degree of coronary artery damage

Our study results showed that NT-proBNP concentration increased with the severity of coronary artery damage, according to the SYNTAX score group. The gradual increase in NT-proBNP concentration was statistically significant (*P* = 0.001). Besides, we also noted that there was a moderately positive correlation between NT-proBNP concentration and the degree of coronary damage according to the SYNTAX score, with *P* < 0.01 and rho = +0.453 (Table 4). Our result was similar to other studies such as Sahin et al. and Magdy et al., which also showed a moderately positive correlation between NT-proBNP concentration and the degree of coronary damage according to the SYNTAX score, with *r* = +0.514 [12] and *r* = +0.443 [13], respectively, with *P* < 0.001. The AUC (ROC) of the NT-proBNP concentration value was 0.75, with a cutoff point of NT-proBNP concentration of 1120.5 pg/mL, predicting an intermediate and high SYNTAX score with 60% sensitivity and 80.3% specificity (Figure 2). Compared with the author, Kurtul et al. noted that the cutoff value of NT-proBNP at 1614 pg/mL had a sensitivity of 75% and a specificity of 68% in predicting the severity of high coronary artery damage according to the SYNTAX score with an AUC (ROC) of 0.761 [14]. Although the underlying pathophysiological mechanism of the association between raised NT-proBNP levels and a higher SYNTAX score in ACS was not fully established in our study, many mechanisms could be at work. One of the more credible explanations could be the severity of myocardial ischemia. Myocardial ischemia causes increased heart-filling pressure, which leads to increased myocardial stretch, which increases NT-proBNP synthesis and release. Furthermore, regardless of hemodynamic circumstances, ischemia or damaged cardiac tissue produces higher NT-proBNP [15]. Furthermore, serum NT-proBNP levels may be a new approach to diagnosing silent myocardial ischemia. Goetze et al.'s findings strongly suggest that acute myocardial hypoxia/ischemia increases BNP expression and the release of freshly synthesized NT-proBNP. In this study, serum NT-proBNP levels were steadily elevated in patients with ACS based on low to high SYNTAX score tertiles, particularly in the myocardial infarction group, which had the most patients in the study [15-17]. We hypothesized that more acute ischemia caused by a higher SYNTAX score would result in higher NT-proBNP levels.

Hs-Troponin T concentration and the degree of coronary artery damage

There was a positive but weak correlation between hs-Troponin T concentration and the extent of coronary artery damage according to the SYNTAX score, with *P* < 0.01 and rho = +0.387 (Table 4). Our study result was lower than the study by Cardoso et al., involving 174 ACS patients, with a positive moderate linear correlation of hs-Troponin T levels with the complexity of coronary artery lesions assessed by the SYNTAX score with a rho of +0.44 [18]. The data collection in our study and the study of Cardoso et al. included patients with unstable angina who did not have increased hs-Troponin T levels, which could contribute to a difference in the mean value of the level of hs-Troponin T in two groups (Table 2).

The AUC (ROC) of the hs-Troponin T concentration value was 0.701, with a cutoff point of the hs-Troponin T concentration of 0.109 ng/mL. This predicted an intermediate and high SYNTAX score with 76% sensitivity and 59% specificity (Figure 1). Compared with the study of author Ucar et al., the AUC (ROC) was 0.86 with a hs-Troponin T concentration value of 0.00962 ng/mL, predicting a higher level of coronary damage of 10.3 points according to the SYNTAX score with a sensitivity of 78.5% and a specificity of 77.7% [19]. On the other hand, a study by Altun et al., involving 287 patients, identified a positive correlation between hs-Troponin T concentration and SYNTAX score, with an *r *value of 0.327. They established a cutoff value for hs-Troponin T of 0.1168 ng/mL to predict a high SYNTAX score, achieving an ROC area of 0.71, 86% sensitivity, and 49% specificity [11]. It could be explained that the study population of Altun et al. comprised patients diagnosed with myocardial infarction, leading to higher hs-Troponin T levels. In contrast, the study population of Ucar et al. consisted of patients with angiographically stable coronary artery disease, resulting in lower hs-Troponin T levels compared to our study.

Limitations

Our study has several limitations that need to be acknowledged. First, this study was a cross-sectional descriptive study with a relatively small number of patients. Second, hs-troponin T and NT-proBNP were measured only on admission. Finally, the assessment of coronary angiography was limited to visual interpretation, and this technique mainly detected major coronary arteries. Further studies with a larger sample size would be needed to have a better understanding of these two markers in the evaluation of coronary artery damage.

## Conclusions

There was a positive moderate correlation between NT-proBNP concentrations and the severity of coronary artery disease according to the SYNTAX score, with *P* < 0.01 and rho = +0.453, whereas there was a positive weak correlation between hs-Troponin T concentrations and the severity of coronary artery disease according to the SYNTAX score, with *P* < 0.01 and rho = +0.387. Hs-Troponin T levels of 0.109 ng/mL exhibited higher sensitivity (76%) but lower specificity (59%) in predicting intermediate and high SYNTAX scores in patients with ACS compared to NT-proBNP levels of 1120.5 pg/mL, which had a sensitivity of 60% and a specificity of 80.3%.
